# Towards stroke prediction using electronic health records

**DOI:** 10.1186/s12911-018-0702-y

**Published:** 2018-12-04

**Authors:** Douglas Teoh

**Affiliations:** Research and Development Group, Allm Inc., Yushin Bldg. Shinkan 2F, 3-27-11 Shibuya, Shibuya-ku, Tokyo, 150-0002 Japan

**Keywords:** Electronic health record, Health data preprocessing, Recurrent neural network, Stroke prediction

## Abstract

**Background:**

As of 2014, stroke is the fourth leading cause of death in Japan. Predicting a future diagnosis of stroke would better enable proactive forms of healthcare measures to be taken. We aim to predict a diagnosis of stroke within one year of the patient’s last set of exam results or medical diagnoses.

**Methods:**

Around 8000 electronic health records were provided by Tsuyama Jifukai Tsuyama Chuo Hospital in Japan. These records contained non-homogeneous temporal data which were first transformed into a form usable by an algorithm. The transformed data were used as input into several neural network architectures designed to evaluate efficacy of the supplied data and also the networks’ capability at exploiting relationships that could underlie the data. The prevalence of stroke cases resulted in imbalanced class outputs which resulted in trained neural network models being biased towards negative predictions. To address this issue, we designed and incorporated regularization terms into the standard cross-entropy loss function. These terms penalized false positive and false negative predictions. We evaluated the performance of our trained models using Receiver Operating Characteristic.

**Results:**

The best neural network incorporated and combined the different sources of temporal data through a dual-input topology. This network attained area under the Receiver Operating Characteristic curve of 0.669. The custom regularization terms had a positive effect on the training process when compared against the standard cross-entropy loss function.

**Conclusions:**

The techniques we describe in this paper are viable and the developed models form part of the foundation of a national clinical decision support system.

## Background

As of 2014, data published by the Statistics Bureau of Japan showed stroke to be the fourth leading cause of death in Japan, with cerebral infarction being the leading cause of stroke [1]. A study on the lifetime risk of stroke in Japan reported observed probabilities of around 1 in 5 middle aged men and women suffering from stroke [2]. When broken down into cause of stroke subtypes, the observed probabilities of being at risk of cerebral infarction are around 1 in 7 for men and 1 in 6 for women. The other causes of stroke are cerebral hemorrhage and subarachnoid hemorrhage. The reported probabilities for cerebral hemorrhage were 1 in 40 for men and 1 in 60 for women, while for subarachnoid hemorrhage, the reported probabilities were 1 in 200 for men and 1 in 50 for women. The lifetime risks were reported to be similar across the studied age groups.

In this paper, we present our attempt at predicting future diagnosis of stroke for patients who have not and may or may not yet suffer from stroke. Applying computational techniques on Electronic Health Records (EHRs) is a common approach for predicting patient outcomes, which is a strategy we have also chosen. Our contributions are as follows. We obtain historical patient EHRs supplied by a hospital. These EHRs contain historical medical diagnoses and exam results. To make use of this data, we perform empirical testing of several pre-processing strategies to determine the best approach for addressing the issues of missing data and use of non-homogeneous and non-numerical data. We then design prediction models using neural network architectures in order to both evaluate the efficacy of the supplied EHR data and the network architecture in our stroke prediction task. Finally, we also present our modifications to the standard cross-entropy loss to include regularization terms, which we show to be useful when training neural networks with imbalanced output classes.

### Related work

Various techniques have been employed to predict the risk of stroke. Cox proportional hazards regression is one technique used to develop a statistical model, where the use of the Framingham Study cohort forms one such application [3]. This application, developed on top of data collected from a sample population over a span of 36 years, described a formula that estimated the probabilities of stroke based on pre-determined risk factors such as age, systolic blood pressure, and presence of diabetes mellitus. Cox proportional hazards regression is commonly used to develop stroke risk models tailored towards a cohort of interest [4, 5]. Globorisk [6] was another model developed with Cox proportional hazards regression, with the aim of producing a formula that can be recalibrated and updated for use in different countries.

Bayesian Rule Lists (BRL) were used to develop an interpretable model to predict risk of stroke within a year for patients diagnosed with atrial fibrillation [7]. BRL produces a hierarchy of decision lists (chains of if …then …rules) ordered by posterior consequent distributions. The model used drug prescriptions, medical conditions, age, and gender to form these decision lists.

Support Vector Machines have been used in a study to predict the occurrence of stroke within five years after a set of baseline measurements [8]. The study also identified issues pertaining to missing data and large number of input features. Missing data was addressed through median imputation after a comparison with other methods. A novel feature selection algorithm was used to reduce the number of input features.

For the diagnosis of other kinds of disorders, or the development of decision support systems, different techniques have also been used. One of the first diagnosis systems was developed in 1961 [9]. This system focused on the diagnosis of congenital heart disease from clinical data, using a diagnostic model derived using Bayes’ theorem. In 1988, the earliest known disease diagnostic system using a multi-layer neural network was developed [10]. This system used over 200 questionnaire responses as inputs and supported 23 diseases as the diagnosis output.

More recently other types of neural network architectures have been investigated for creating predictions from EHRs. Doctor AI [11] employed a Recurrent Neural Network (RNN) architecture to process patient temporal medical events and prescriptions, which were both represented as categorical features. The model was used to predict future diagnoses, medication orders and visit time.

ICD-9 label assignments can be used to classify medical notes using a bag-of-words model combined with RNN [12]. The methods used to represent medical notes has a demonstrable effect on performance when evaluated on event prediction tasks such as patient mortality and emergency room visits [13].

On the topic of EHR representation, Deepr [14] drew on natural language processing techniques to represent diagnoses and treatments as a sequence of tokens. Temporal features were also discretized and represented as tokens. These tokens were then transformed into a continuous vector space through an embedding process. This representation was fed into a Convolutional Neural Network (CNN) architecture and evaluated on its ability to predict future hospital readmission risk.

Autoencoders can be used to derive compressed representations of physiological features as an input pre-processing step [15]. The word2vec algorithm [16] can be applied to generate an embedding over patient diagnoses and medications [17]. The embedded representation was fed into a CNN, whereby event temporality was handled with 1-dimensional convolutions over the temporal dimension of the input matrix.

## Methods

In this section, we describe our data source, formally identify and formulate the problem we want to solve, and describe the machine learning models that we implemented in an attempt to solve the stroke prediction task.

### Data source

Our data was supplied by Tsuyama Jifukai Tsuyama Chuo Hospital in Okayama Prefecture, Japan.

#### Patient cohort

The patient cohort was divided into two groups: case patients and control patients. Case patients are patients who will be diagnosed with stroke. The criteria for determining case patients were as follows. The initial set of case patients had a first diagnosis of stroke between January 1, 2001 and January 1, 2015. The specific date range was chosen arbitrarily, with the intention of accounting for latent variables which could include access to newer drugs, changes in data recording policies, and even socioeconomic changes. The patient cohort was further filtered to include only those who received the first diagnosis of stroke between the ages of 45 and 95. This range was chosen because there were at least 10 incidents of stroke per age. Case patients were also selected to have at least one medical diagnosis and at least one exam result at least one day before the stroke diagnosis.

To maximize both the number of case and control patients, only a maximum of two control patients were able to be selected per case patient. To be selected as a control patient, the candidate must have the same birth year and gender as the case patient, at least one medical diagnosis and one exam result before the date the case patient was diagnosed with stroke, and at least one medical diagnosis and one exam result after the date the case patient was diagnosed with stroke. Control patients also did not have any diagnosis of stroke in their EHR. Case patients without two control patients were excluded from the final set of case patients.

After applying these criteria, 2725 case patients and 5450 control patients were identified. These patients formed the dataset used for training and evaluating our models.

#### Patient diagnoses

Each diagnosis was recorded in the EHR as a tuple comprising of the diagnosis date and the ICD-10 code [18] representation of the diagnosis. For this study, codes longer than four letters were truncated to be four letters long. The use of codes longer than four letters comprised around 5% of diagnoses across all patients. In fact, five letter codes were the longest codes in the hospital’s EHR system. The truncation posed no problem because ICD-10 codes form a prefix hierarchy. We must also note that ICD-10 codes were derived through a mapping from Japanese insurance codes to ICD-10 codes of the year 2013 edition. Our study supported a total of 6905 ICD-10 codes. Stroke cases were identified by the range of codes spanning I60 to I69.

#### Patient exam results

We were supplied with blood and biochemical exam results for each patient. Each exam was comprised of one or more measured metrics, but an exam did not necessarily contain the same set of measured metrics. For example, a blood exam could contain results for red blood cell (RBC) count but not every blood exam was guaranteed to contain results for RBC count. There can also be two measurements per metric. The blood exam metrics were: (a) ALP (b) creatinine (c) Hb (d) hematocrit (e) MCH (f) MCHC (g) MCV (h) platelet count (i) RBC and (j) WBC. The biochemical exam metrics were: (a) GGT (b) GOT (c) GPT (d) HbA1c JDS (e) HbA1c NGSP (f) HDL-C (g) LDL-C (h) triglyceride (i) uric acid and (j) blood glucose.

We experimented with a few strategies to account for missing or optional data. We tried adding a categorical value per metric to indicate if the measurement was absent, as a means of differentiating zero values from unrecorded values. Our models did not have any predictive ability when this strategy was used. We tried a roll-forward strategy, where each missing value was replaced with the last known recorded value. Our models also failed to learn with this strategy. We then tried augmenting the roll-forward strategy by replacing missing values with the mean measured value for the patient’s gender using data from the training set, and our models were able to start learning with this strategy. Finally, to address the differences in the number of exam results between patients, exam metrics were summarised by age, i.e. keep only the newest recorded value for each metric at a given age. We found that our models attained the best results with this strategy.

### Problem description

The purpose of this study was to investigate the possibility of predicting whether a patient will suffer from stroke within a year of the last set of exam results or the last set of medical diagnoses. A period of a year was chosen because annual health exams are mandatory for full time employees in Japan [19]. Being able to predict future illness from a regular exam would enable proactive forms of healthcare measures to be taken.

We formulated the prediction task as a binary classification task. The inputs available to this task were a patient’s historical exam results ***X***_*exam*_ and medical diagnoses ***X***_*diagnosis*_. The output $\hat {\boldsymbol {y}}$ was a yes/no decision to indicate if the patient would suffer from stroke in the next 365 days. The following models were implemented and evaluated:

1 $$\begin{array}{*{20}l} \hat{\boldsymbol{y}} &= f_{d}\left(\boldsymbol{X}_{diagnosis}^{(i)}\right)  \end{array} $$

2 $$\begin{array}{*{20}l} \hat{\boldsymbol{y}} &= f_{e}\left(\boldsymbol{X}_{exam}^{(j)}\right)  \end{array} $$

3 $$\begin{array}{*{20}l} \hat{\boldsymbol{y}} &= f_{ed}\left(\boldsymbol{X}_{diagnosis}^{(i)}, \boldsymbol{X}_{exam}^{(j)}\right)  \end{array} $$

We wanted our models to be able to parameterize by itself, the input features which are useful as predictors for the stroke prediction task. To do this we used all features that were available in the dataset, and encoded them as follows.

The input matrix $\boldsymbol {X}_{diagnosis}^{(i)}$ represented a patient’s *h*^th^ up to the *i*^th^ historical diagnoses:

4 $$ \boldsymbol{X}_{diagnosis}^{(i)} = \left(\boldsymbol{x}_{diagnosis}^{(h)}, \dots, \boldsymbol{x}_{diagnosis}^{(i)}\right) \mid h = \text{max}(1, i - 34), h \ne i  $$

The maximum number of diagnoses in ***X***_*diagnosis*_ was limited to 35 because this covered 99% of patients in the dataset. At least half of the patients had 5 or more diagnoses. There was a long tail of patients having more than 35 diagnoses.

A patient’s *i*^th^ diagnosis was represented by the vector $\boldsymbol {X}_{diagnosis}^{(i)}$. The patient’s gender was encoded as *g*∈{0,1}. To account for the patient’s age at time of diagnosis, this value was included and denoted as $a_{d}^{(i)} \in \mathbb {R}$. Each diagnosis ICD-10 code was encoded into a dense vector $\boldsymbol {d}^{(i)} \in \mathbb {R}^{32}$; details of this encoding process will be described in the next section. The number of days *n* since the (*i*−1)^th^ diagnosis was converted into a categorical value *n*_*c*_ and then encoded into a one-hot label vector $\boldsymbol {c}_{d}^{(i)} \in \{0,1\}^{|n_{c}|}$. The categories were:

5 $$  n_{c} =\left\{ \begin{array}{ll} 0 & \text{if } n \text{ is unknown or the first record}\\ 0 & 0 \leq n < 7\\ 1 & 7 \leq n < 30\\ 2 & 30 \leq n < 90\\ 3 & 90 \leq n < 180\\ 4 & 180 \leq n \leq 365\\ 5 & 365 < n\\ \end{array}\right.  $$

The complete vector representation of the *i*^th^ diagnosis was then formulated as $\boldsymbol {x}_{diagnosis}^{(i)} = \left ((g), \left (a_{d}^{(i)}\right), \boldsymbol {c}_{d}^{(i)}, \boldsymbol {d}^{(i)}\right)$.

The input matrix $\boldsymbol {X}_{exam}^{(j)}$ represented the patient’s first to *j*^th^ summarized exam results:

6 $$  \boldsymbol{X}_{exam}^{(j)} = \left(\boldsymbol{x}_{exam}^{(1)}, \boldsymbol{x}_{exam}^{(2)}, \dots, \boldsymbol{x}_{exam}^{(j)}\right) \mid 0 < j \leq 20  $$

No patient in the dataset had more than 20 years of exam results, so $\boldsymbol {X}_{exam}^{(j)}$ will contain at most 20 results. At least half of the patients had 5 years or more of exam results.

The patient’s *j*^th^ exam results was represented by the vector $\boldsymbol {X}_{exam}^{(j)}$. The patient’s gender was encoded as *g*∈{0,1}. To account for the patient’s age when the exam was conducted, this value was included and denoted as $a_{e}^{(j)} \in \mathbb {R}$. The number of days *n* since the *j*−1^th^ exam was conducted was converted into a categorical value *n*_*c*_ as described in Eq. 5 and then encoded into a one-hot label vector $\boldsymbol {c}_{e}^{(j)} \in \left \{0,1\right \}^{|n_{c}|}$. Each exam metric’s measurement *m* was represented as $e_{m}^{(j)} \in \mathbb {R}$. All exam metrics were combined to form the vector $\boldsymbol {e}_{results}^{(j)} = \left (e_{1}^{(j)}, \dots, e_{m}^{(j)}\right)$. The complete vector representation of the *j*^*th*^ exam was formulated as $\boldsymbol {x}_{exam}^{(j)} = \left ((g), \left (a_{e}^{(j)}\right), \boldsymbol {c}_{e}^{(j)}, \boldsymbol {e}_{results}^{(j)}\right)$.

### ICD-10 encoding

We encoded the ICD-10 codes into the dense vector ***d***. Generally, *N* categorical values can be encoded as a one-hot label vector {0,1}^1×*N*^, where a value of 1 in the *i*^*th*^ column and zero in every other column is a vector representing the *i*^th^ category. This encoding scheme works when the value of *N* does not cause memory usage issues during an algorithm’s runtime. For large *N*, it may be necessary to apply dimensionality reduction techniques to project the input space into a lower dimensional space. We used a single hidden layer autoencoder network to project from the sparse input vector ***x***_*ICD*_∈{0,1}^6905^ into a dense vector $\boldsymbol {d} \in \mathbb {R}^{32}$. Our autoencoder network was defined as

7 $$\begin{array}{*{20}l} \boldsymbol{d} &= \text{tanh}\left(\boldsymbol{x}_{ICD}^{T}\boldsymbol{W}_{input} + \boldsymbol{b}_{input}\right)  \end{array} $$

8 $$\begin{array}{*{20}l} \boldsymbol{y}_{decoding} &= \text{softmax}\left(\boldsymbol{d}^{T}\boldsymbol{W}_{output} + \boldsymbol{b}_{output}\right) \end{array} $$

The autoencoder network was then trained to learn ***W***_*input*_, ***b***_*input*_, ***W***_*output*_, and ***b***_*output*_ such that the categorical cross-entropy loss between ***y***_*decoding*_ and ***x***_*ICD*_ was minimized. Equation 7 can then be applied to yield our dense representation ***d***. Our autoencoder network learned a perfect reconstruction.

### Model implementations

All neural network models presented in this paper were selected through experimentation. These models were selected by evaluating the F_1_ score achieved on the validation set. The experimentation (which included architecture searches and meta-parameter selections) was non-exhaustive; the models presented here make no claims of being globally optimal with respect to predictive power. Our experiments were conducted on a desktop-class machine with a single Nvidia GTX 1080 video card. We spent around 3 months conducting experiments, with the runtime for each experiment ranging from a day up to a week. We present our model descriptions as Keras 2.0.9 [20] code in the interest of being precise.

Model 1 was implemented as a RNN using a Gated Recurrent Unit (GRU) module for recurrent connections. Text translation tasks, such as those described in [21], have demonstrated the capability of RNNs to learn from variable-length sequences and model dependencies within the input sequence. GRU modules were chosen over Long Short Term Memory modules because empirical evidence have demonstrated it to be faster to train while achieving comparable performance [22]. Listing 1 shows the model’s code definition.


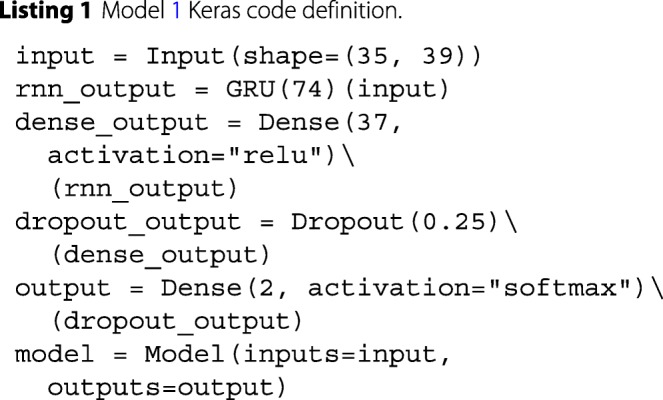


Model 2 was implemented using a fully connected architecture. We experimented with CNN and RNN architectures but did not achieve better performance. We handled temporal input using Keras’ TimeDistributed wrapper. The code for this model is in Listing 2.


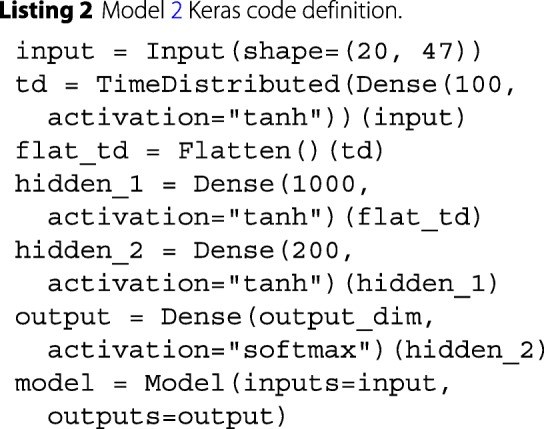


Finally, Model 3 was implemented using a dual-input architecture to handle both diagnoses and exam results as input data. This model was a fusion of Models 1 and 2. Each type of input was processed by a separate branch in the network and the outputs were then concatenated together and processed by the rest of the network. To speed up the training of this model, we transferred weight values from the original neural network models of layers sharing the same configuration. The code for this model is in Listing 3. For clarity, we have omitted the code for overriding the initial weights with transferred weights.


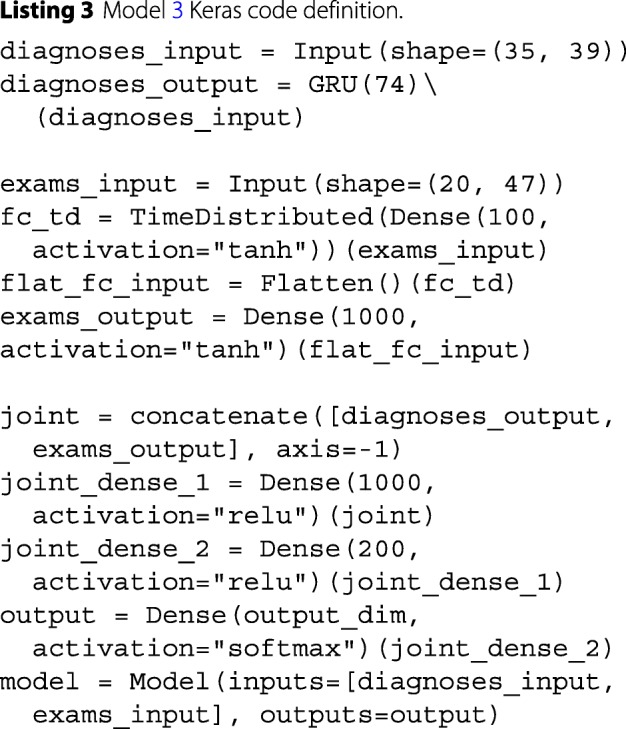


Our dataset was split into training, validation, and test sets at a ratio of 70/15/15. The split was performed by first dividing case patients into the aforementioned subsets. To ensure that each split has the same ratio of case to control patients, control patients who were associated with a case patient became members of the associated case patient’s subset split. Keeping the ratios consistent across each split was crucial when evaluating model performance.

All exam result values were scaled by subtracting the mean and then dividing by the standard deviation. The mean and standard deviation values were calculated from the training set. The prevalence of stroke diagnosis was 1 out of 8 ***X***_*diagnosis*_ inputs and 1 out of 9 ***X***_*exam*_ inputs.

All models were trained to minimize a customised categorical cross-entropy loss. Adam optimizer [23] with default parameters supplied by Keras was used. The random seed was fixed for all models. We used mini-batches of size 32 and trained for 500 epochs. Model 3 was trained for 100 epochs.

Our loss function enhanced the standard categorical cross-entropy loss by incorporating penalties for false positive and false negative predictions. Let *Y*^+^ be positive labels and *Y*^−^ be negative labels. The total number of false positives *fp* and false negatives *fn* can be stated in the terms of the zero-one loss

9 $$ fp(f) = \sum_{i \in Y^{-}} \ell_{01}(f, x_{i}) \qquad \qquad fn(f) = \sum_{i \in Y^{+}} \ell_{01}(f, x_{i})  $$

Then, a suitable surrogate for the zero-one loss can be used to place an upper bound on these quantities and also to make these quantities optimizable through stochastic gradient descent methods [24]. We used log loss as the surrogate since we wanted to incorporate these quantities into the standard categorical cross-entropy loss. These quantities satisfied the following inequalities

10 $$ \begin{aligned} fp^{u}(f) &\triangleq \sum_{i \in Y^{-}} \ell_{ll}(f, x_{i}) \geq fp(f) \\ fn^{u}(f) &\triangleq \sum_{i \in Y^{+}} \ell_{ll}(f, x_{i}) \geq fn(f) \end{aligned}  $$

Where *ℓ*_*ll*_(*f*,*x*)=−*p*(*x*) log*f*(*x*). These quantities were incorporated into the standard cross-entropy loss as

11 $$ \ell(f) = \alpha\left(\sum_{i \in Y} \ell_{ll}(f, x_{i}) \right) + \beta fp^{u}(f) + \gamma fn^{u}(f)  $$

The terms *α*, *β* and *γ* were used to change the influence of each loss term. Our training hyperparameters used *α*=0.2,*β*=5 and *γ*=5.

## Results

### Model performance

We illustrate the performance of our models using the Receiver Operating Characteristic (ROC) curve which displays the trade-off between sensitivity and specificity at each threshold. ROC curves are commonly used in medical decision making [25]. Sensitivity is defined as the sum of true positive predictions divided by the total population of positive conditions. Specificity is defined as the sum of true negative predictions divided by the total population of negative conditions.

To assist in the comparison of our models, we present the area under the ROC curve (AUC) value, which is the probability of ranking a randomly chosen positive instance higher than a randomly chosen negative instance. A better-than-random model will have an AUC value greater than 0.5, while a perfect model will have an AUC value of 1.0. [25]

Finally, we also include the 95% confidence interval of the ROC curves calculated through bootstrapping and stratified sampling. All ROC curves presented in this paper were generated using pROC [26].

**Fig. 1:**
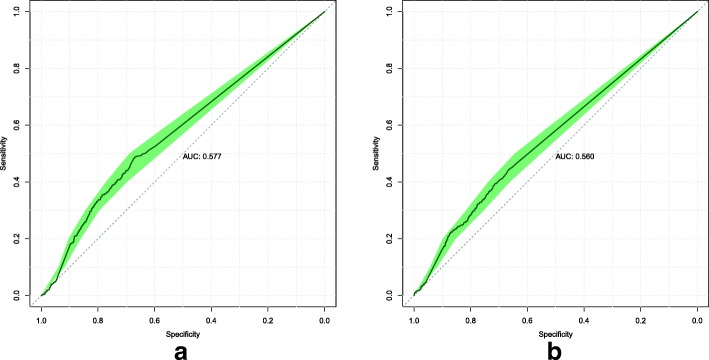
Model 1 ROC curves attained on the validation (**a**) and test (**b**) sets. The shaded area displays the 95% confidence interval. The AUC is calculated from the solid line

**Fig. 2:**
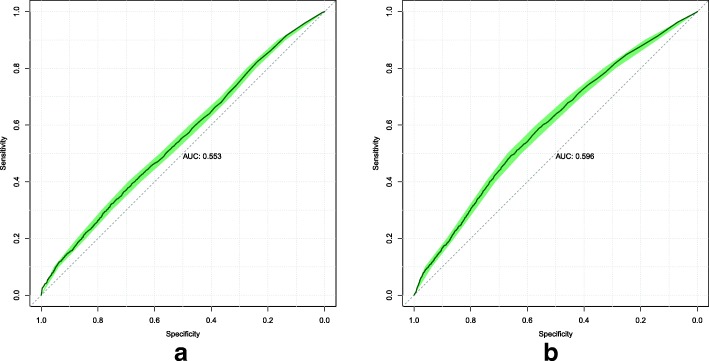
Model 2 ROC curves attained on the validation (**a**) and test (**b**) sets. The shaded area displays the 95% confidence interval. The AUC is calculated from the solid line

**Fig. 3:**
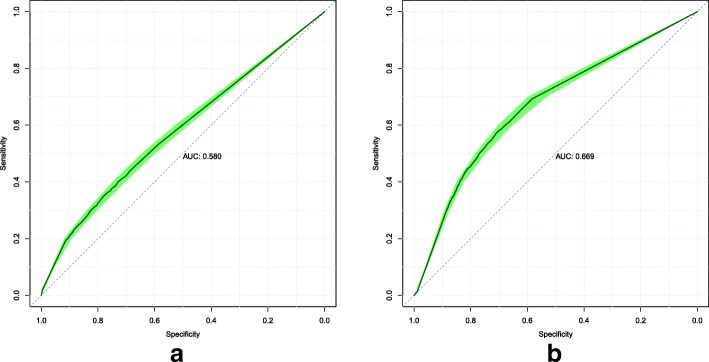
Model 3 ROC curves attained on the validation (**a**) and test (**b**) sets. The shaded area displays the 95% confidence interval. The AUC is calculated from the solid line

The ROC curves were computed for each model on the validation and test sets. Model 1’s ROC curves are displayed in Fig. 1. Model 2’s ROC curves are displayed in Fig. 2. Model 3’s ROC curves are displayed in Fig. 3. We summarize the performance of each model as being better-than-random, with Model 3 attaining the best overall predictive power when measured by AUC on both the validation and test sets.

### Custom loss function

**Fig. 4:**
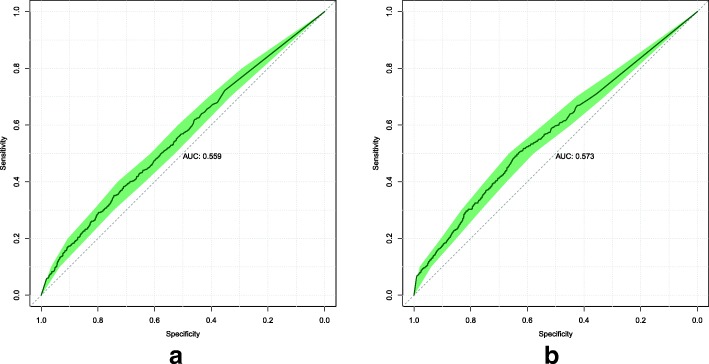
Model 1 ROC curves attained on the validation (**a**) and test (**b**) sets, when trained to minimize the standard cross-entropy loss. The shaded area displays the 95% confidence interval. The AUC is calculated from the solid line

**Fig. 5:**
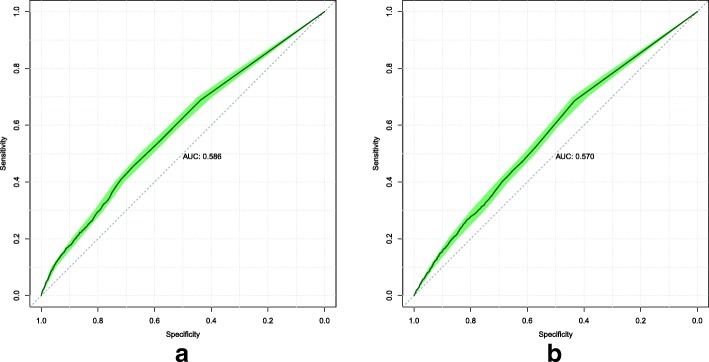
Model 2 ROC curves attained on the validation (**a**) and test (**b**) sets, when trained to minimize the standard cross-entropy loss. The shaded area displays the 95% confidence interval. The AUC is calculated from the solid line

We conducted an ablation study to determine the efficacy of the custom loss function described in Eq. 11. For all models, we kept the same neural network architecture and training methodology but changed the loss function to the standard cross-entropy loss. Model 1’s ROC curves are displayed in Fig. 4. Model 2’s ROC curves are displayed in Fig. 5.

**Fig. 6:**
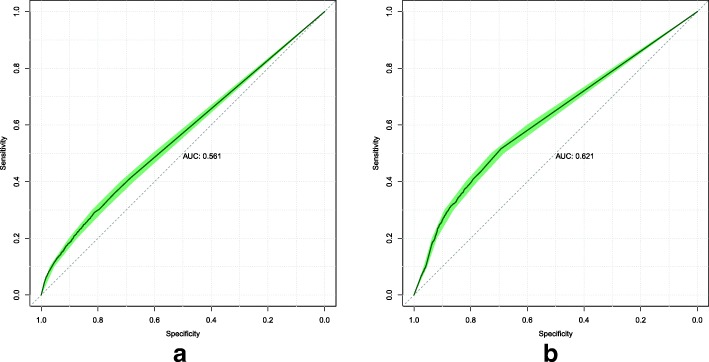
Model 3 ROC attained on the validation (**a**) and test (**b**) sets, when initialized with weights transferred from models trained with the custom loss function, and then trained to minimize the standard cross-entropy loss. The shaded area displays the 95% confidence interval. The AUC is calculated from the solid line

**Fig. 7:**
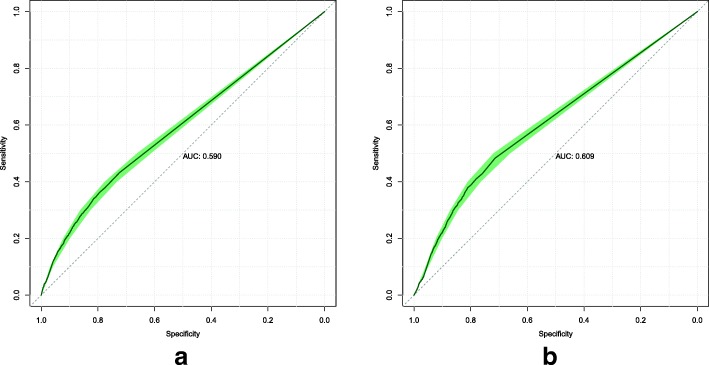
Model 3 ROC attained on the validation (**a**) and test (**b**) sets, when initialized with weights transferred from models trained with the standard cross-entropy loss function, and then trained to minimize the standard cross-entropy loss. The shaded area displays the 95% confidence interval. The AUC is calculated from the solid line

To evaluate Model 3, we tested two weight transfer configurations. The first configuration tested weights transferred from models trained with the custom loss function; the ROC curves are displayed in Fig. 6. The second configuration tested weights transferred from models trained with the standard loss function; the ROC curves are displayed in Fig. 7.

### Exam importance

To determine which exams were considered to be important by our trained model, we conducted ablation studies using Model 3. For these studies, we combined the validation and test sets and calculated the AUC attained when an exam (comprised of one or more exam metrics) was excluded from the input to the model. We excluded the exam by setting the value of associated metrics to zero. The results are displayed in Table 1.

**Table 1:** Impact on predictive performance of exams on Model 3

Model Inputs	AUC	95% CI
with everything	0.623	(0.614, 0.632)
w/o Cholesterol Exam (HDL-C, LDL-C, triglyceride)	0.618	(0.610, 0.627)
w/o Diabetes Exam (HbA1c JDS & NGSP, blood glucose)	0.608	(0.600, 0.617)
w/o Kidney Exam (creatinine)	0.624	(0.615, 0.632)
w/o Liver Exam (GGT, GOT, GPT)	0.615	(0.606, 0.623)
w/o Platelet count	0.607	(0.599, 0.616)
w/o ALP	0.616	(0.607, 0.625)
w/o Blood count (Hb, hematocrit, MCH, MCHC, MCV, RBC)	0.600	(0.591, 0.609)
w/o Uric acid	0.627	(0.618, 0.635)
w/o WBC	0.608	(0.600, 0.617)

w/o = without

## Discussion

### Model performance

The models that were implemented all demonstrate better-than-random predictive power. This shows the viability of using data already present in EHRs to predict a future diagnosis of stroke. In this study, we only had diagnoses and exam results data. To determine which of these two data types would have more predictive power, we can compare the performance of Models 1 and 2. On the validation set, we find that the diagnoses-only model attained better performance (AUC 0.577 vs 0.553, from Figs. 1 and 2 respectively). On the test set, Model 2 attained better performance (AUC 0.596 vs 0.560, from Figs. 2 and 1 respectively). Based on this, we think that the predictive power of both diagnoses and exam results data are roughly similar.

The performance attained by Model 3 shows the value of integrating diagnoses and exam results data into a single model. The result on the test set (AUC 0.669 from Fig. 3) shows a clear performance improvement over Models 1 and 2. This suggests that the neural network was able to exploit relationships between diagnoses and exam results to deliver better predictions. We think that sourcing more data types, such as qualitative (e.g. smoking habits, sleep quality) or non health-related (e.g. income) data, to integrate together could deliver even better predictions.

### Custom loss function

The results of the custom loss function’s efficacy when applied to Models 1 and 2 are mixed. For Model 1, the custom loss function is better on the validation set by around 0.018 AUC, but worse on the test set by around 0.013 AUC. For Model 2, the reverse is true. The standard loss function is better on the validation set by around 0.033 AUC, but worse on the test set by around 0.026 AUC.

For Model 3, the results show the custom loss function to be better in all cases except for one. The model initialized with weights transferred from models trained with the standard cross-entropy loss was better by around 0.01 AUC on the validation set. However, the custom loss function achieved better performance on the test set by around 0.06 AUC. When comparing the results for the standard cross-entropy loss model initialized with weights transferred from models trained with the custom loss function, the custom loss function was better by around 0.019 AUC and 0.048 AUC on the validation and test sets respectively.

Overall, the custom loss function demonstrated performance improvements in most cases. We think that the custom loss function has a larger impact when used with more complex models. We hypothesize that this is because the weighted surrogate quantities for false positive and false negative predictions introduced useful regularization terms to the standard cross-entropy loss. These regularization terms enabled our more complex network architecture to attain better results by attaching large penalties to false positive and false negative predictions. Since the prevalence of negative predictions outweigh positive predictions, future work could investigate changing the weights so that false negative predictions are penalized more than false positive predictions. We can conclude that the custom loss function is useful when training neural networks on binary prediction tasks with imbalanced classes.

### Exam importance

From the results displayed in Table 1, the top three exams or inputs having the most effect on model performance are blood count, platelet count, and the diabetes exam. The inputs for the blood count exam (comprising of Hb, hematocrit, MCH, MCHC, MCV, and RBC) has the most impact on model performance. When removed, the model’s AUC dropped from 0.623 to 0.600. Removing the platelet count input caused the AUC to drop from 0.623 to 0.607. Removing the diabetes exam inputs (comprising of HbA1c JDS, HbA1c NGSP, and blood glucose) caused the AUC to drop from 0.623 to 0.608.

To understand the significance of these findings, we investigated medical research literature for supporting evidence. Hb level at time of acute ischemic stroke has been found to be associated with larger infarcts and increased infarct growth [27]. Higher red blood cell distribution width values (calculated from MCV [28]), was found to have a relation to stroke occurrence and may be a possible predictor of future cardiovascular mortality among persons with a history of stroke [29]. The increase in platelet count has been found to be associated with ischemic stroke, while a decrease in platelet has been found to be associated with hemorrhagic stroke [30]. Some studies have found an association between higher HbA1c levels and increased risk of stroke and cardiovascular disease [31, 32].

While we have not conducted a thorough review of medical literature, we think that our trained model has demonstrated that it is making predictions that has some foundation in contemporary medical findings.

### Limitations

The contents of our dataset did not include all risk factors for stroke identified by other studies [3, 6]. Some of these missing risk factors include but are not limited to systolic blood pressure, smoking habits, treatment for hypertension, and electrocardiogram data. We could not include this data because it was either not available, available but not in sufficient quantities, or available but withheld from distribution. As a result of not having suitable data for these risk factors, faithful comparisons against other published models and results, such as the Cox proportional hazards regression model, were not performed. A proper comparison necessitates replicating published models with data available in our study, and then reporting on those results. These models are commonly used by physicians to assess patient risk; without replicating those baseline models we do not currently make claims of being better than physicians. On the other hand, our models use data not considered by those baseline models while delivering predictive performance. We think that this shows value in incorporating data not used by standard models.

## Conclusion

Accurate stroke prediction remains an unsolved problem. However, this paper has presented a working neural network model which serves as a starting point in what will be an ongoing research topic. Along the way we developed a working regularization technique for dealing with the low prevalence of stroke cases when generating predictions for a bounded future timeframe. Low prevalence of a particular condition or diagnosis is a common attribute of medical prediction tasks, and the regularization technique we have presented is easily incorporated to improve performance.

Future research would begin with an expansion of the dataset to source more case and control patients, and to also include the risk factors being evaluated by other stroke prediction models. Sourcing more patients would potentially allow the experimental setup of case and control patients to be closer in form to that of a cohort study. Finally, the presented models could be further improved through architectural changes or by incorporating advances in machine learning research.

The work presented in this paper forms part of the foundation of a national (and potentially global) clinical decision support system. A system like this can be applied in areas such as preventive medicine, insurance forecasting, and personalized healthcare. We think that such a system will provide a net benefit to society in general.

## Abbreviations

ALP: Alkaline phosphatase
AUC: Area under the receiver operating characteristic curve
BRL: Bayesian rule lists
CNN: Convolutional neural network
EHR: Electronic health record
GGT: Gamma-glutamyl transferase
GOT: Glutamic-oxaloacetic transaminase
GPT: Glutamic-pyruvic transaminase
GRU: Gated recurrent unit
HDL-C: High-density lipoprotein cholesterol
HbA1c: Glycated hemoglobin
Hb: Hemoglobin
ICD-9: 9th revision of the international statistical classification of diseases and related health problems
ICD-10: 10th revision of the international statistical classification of diseases and related health problems
JDS: Japan diabetes society
LDL-C: Low-density lipoprotein cholesterol
MCHC: Mean corpuscular hemoglobin concentration
MCH: Mean corpuscular hemoglobin
MCV: Mean corpuscular volume
NGSP: National glycohemoglobin standardization program
RBC: Red blood cell
RNN: Recurrent neural network
ROC: Receiver operating characteristic
WBC: White blood cell
